# An Integrated Approach to Elucidate the Interplay between Iron Uptake Dynamics and Magnetosome Formation at the Single-Cell Level in *Magnetospirillum gryphiswaldense*

**DOI:** 10.1021/acsami.4c15975

**Published:** 2024-10-31

**Authors:** Marta Masó-Martínez, Josh Bond, Chidinma A Okolo, Archana C Jadhav, Maria Harkiolaki, Paul D Topham, Alfred Fernández-Castané

**Affiliations:** † Energy and Bioproducts Research Institute, Aston University, Birmingham B4 7ET, United Kingdom; ‡ Aston Institute for Membrane Excellence, Aston University, Birmingham B4 7ET, United Kingdom; § Beamline B24, Diamond Light Source, Harwell Science and Innovation Campus, Didcot, Oxfordshire OX11 0DE, United Kingdom; ∥ Chemistry Department, University of Warwick, Coventry CV4 7SH, United Kingdom

**Keywords:** biomineralization, magnetic nanoparticles, magnetotactic bacteria, correlative microscopy, soft X-ray tomography, magnetosomes, cryo-structured illumination microscopy

## Abstract

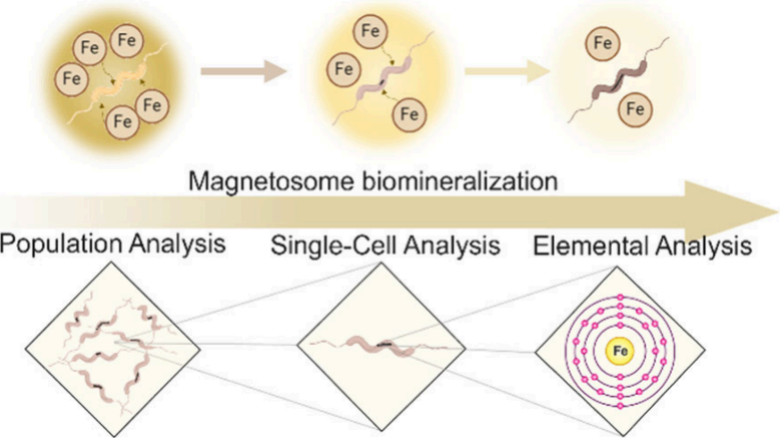

Iron is a crucial element integral to various fundamental biological molecular mechanisms, including magnetosome biogenesis in magnetotactic bacteria (MTB). Magnetosomes are formed through the internalization and biomineralization of iron into magnetite crystals. However, the interconnected mechanisms by which MTB uptake and regulate intracellular iron for magnetosome biomineralization remain poorly understood, particularly at the single-cell level. To gain insights we employed a holistic multiscale approach, *i*.*e*., from elemental iron species to bacterial populations, to elucidate the interplay between iron uptake dynamics and magnetosome formation in *Magnetospirillum gryphiswaldense* MSR-1 under near-native conditions. We combined a correlative microscopy approach integrating light and X-ray tomography with analytical techniques, such as flow cytometry and inductively coupled plasma spectroscopy, to evaluate the effects of iron and oxygen availability on cellular growth, magnetosome biogenesis, and intracellular iron pool in MSR-1. Our results revealed that increased iron availability under microaerobic conditions significantly promoted the formation of longer magnetosome chains and increased intracellular iron uptake, with a saturation point at 300 μM iron citrate. Beyond this threshold, additional iron did not further extend the magnetosome chain length or increase total intracellular iron levels. Moreover, our work reveals (i) a direct correlation between the labile Fe^2+^ pool size and magnetosome content, with higher intracellular iron concentrations correlating with increased magnetosome production, and (ii) the existence of an intracellular iron pool, distinct from magnetite, persisting during all stages of biomineralization. This study offers insights into iron dynamics in magnetosome biomineralization at a single-cell level, potentially enhancing the industrial biomanufacturing of magnetosomes.

## 1 Introduction

Iron constitutes an essential element in all living organisms due to its participation in a wide range of fundamental biological processes such as respiration, nitrogen fixation and DNA synthesis.^1^ Particularly, iron plays a crucial role in a group of Gram-negative bacteria known as magnetotactic bacteria (MTB). These bacteria biomineralize large amounts of iron (up to 4% of their dry weight) to form a type of magnetic nanoparticles called magnetosomes in a complex metabolic process.

Magnetosomes are nanoscale organelles consisting of membrane-coated magnetic crystals of magnetite (Fe_3_O_4_) or greigite (Fe_3_S_4_) usually arranged as a needle-like chain and their main function is to act as geomagnetic navigational systems.^2^ Magnetosomes have tremendous potential for biotechnological and biomedical applications due to their unique properties such as narrow size distribution and biocompatibility.^3^ However, their industrial biomanufacturing potential is hindered by their relatively limited growth and low magnetosome production yields.^4,5^ This limitation is likely due to the insufficient understanding of the magnetosome biomineralization process at the single-cell level, which may involve several factors, such as intrinsic energetic constraints (e.g., ATP generation/consumption), nutrient availability, the influence of secondary metabolites, or environmental conditions.^4^ Addressing these knowledge gaps could help unlock the full production potential of magnetosomes.

Despite the efforts invested in understanding the metabolic and biochemical pathways responsible for magnetite biomineralization, the mechanisms by which MTB uptake iron from the environment, its intracellular storage, and internalization into the magnetosome vesicle remain poorly understood. Several models have been proposed. The first model suggests that iron is directly transported into magnetosome vesicles from the periplasm when the vesicle lumen is still in contact with the cytoplasmatic membrane by direct transport or diffusion.^6^ The second model proposes intracellular accumulation of Fe^2+^ and ferritin and subsequent transportation to the magnetosome vesicle for its coprecipitation into magnetite.^7^ Finally, the third model suggests that Fe^2+^ and Fe^3+^ are transported through the cytoplasmatic membrane via general iron uptake systems, such as the Feo (ferrous iron transport) system^8,9^ and transported into magnetosome vesicles with the help of magnetosome-specific transporters (MamB, MamM, MamH, MamZ)^2,10,11^ with or without the help of ferric reductases that convert Fe^3+^ to soluble Fe^2+^.^12,13^

Previous transmission electron microscopy (TEM) analyses combined with X-ray absorption spectroscopy and Mössbauer spectroscopy studies revealed the presence of other iron species besides magnetite involved in early stages of magnetite biogenesis (e.g., ferrihydrite, ferritin proteins, hematite).^7,14−17^ This has led to the hypothesis that 99% of the intracellular iron corresponds to magnetite, as most of these distinct iron species were no longer detected in the intracellular iron pool by the end of the biomineralization process. However, recent studies demonstrated that magnetite constitutes only 25–45% of the total intracellular iron content,^18−21^ evidencing the heterogeneity of the intracellular iron pool in MTB.

The use of single-cell analysis tools has yielded valuable information regarding the intracellular iron pool and magnetosome biomineralization.^20^ For instance, fluorescence microscopy has been used to characterize intracellular iron speciation and subcellular localization;^19,22^ flow cytometry (FCM) to monitor physiological parameters, including the intracellular iron pool;^23^ and single-cell inductively coupled plasma mass spectroscopy (ICP-MS) to quantify the intracellular iron content of individual MTB cells.^24^ Despite providing valuable information about the intracellular iron pool, these techniques are limited in their ability to measure magnetosome content at the single-cell level. In contrast, techniques that can quantify magnetosome content at the single-cell level (e.g., TEM) cannot measure the intracellular iron pool.^25^ Therefore, using a combination of these techniques compensates for the limitations of each method. For example, FCM is a high-throughput technique that enables real-time single-cell analysis of thousands of cells within a brief time frame, examines heterogeneous cell populations within a single sample, and provides physiological information such as cell size and complexity.^26^ However, FCM only provides relative measurements of cellular characteristics but lacks detailed morphological information compared with microscopic techniques. Bulk quantification of magnetosome content is typically done by ICP-optical emission spectroscopy (ICP-OES) or other spectroscopic techniques (e.g., AAS) and supported by TEM magnetosome visualization.^27^ Both methods require offline measurements, impeding real-time data acquisition while performing the experiment and involving tedious sample preparation procedures. TEM analysis typically involves sample drying, staining, and sectioning, potentially inducing sample damage and compromising the visualization of cells under their native conditions, which may influence the resultant data.^28^

Here, we propose for the first time the utilization of a correlative microscopy approach involving cryo-structured illumination microscopy (cryoSIM) and synchrotron-based cryo-soft X-ray tomography (cryoSXT)^29^ on *Magnetospirillum gryphiswaldense* MSR-1, alongside the use of FCM, ICP-OES, and TEM to enhance our understanding of this intracellular iron pool and its relationship with magnetosome synthesis at single-cell level under different oxygen and iron dosage regimes. CryoSIM was employed to obtain information on the labile Fe^2+^ intracellular pool, utilizing a fluorescent iron probe, and cryoSXT enabled the acquisition of structural cell information and the characterization of the magnetosome content for each cell. Another important advantage of using a technique such as cryoSXT, where samples are cryo-fixed, is the preservation of their native-hydrated state, which is crucial for obtaining valuable structural and functional information. Although other correlative cryo-imaging techniques, such as the combination of cryo-fluorescence and cryo-electron microscopy, are widely used to study relevant cellular structures and dynamic processes such as bacteria-cell interactions,^30^ the significant resolution gap between visible light and electron microscopy can pose challenges in data correlation. CryoSXT therefore bridges the gap between these two techniques as a mesoscale imaging method, occupying this resolution gap and facilitating more effective data integration.^31^ This combinatorial toolkit of quantitative and qualitative methodologies spans multiple scales, from population analysis and observation of physiological cell behaviors to elemental analysis and examination of individual cells. The combined application of these techniques mitigates the inherent limitations through the strengths of other complementary methods. This strategy has led to the acquisition of more robust data sets and yields valuable insights that might otherwise have gone unnoticed.

Overall, the use of this holistic multiscale approach not only allows a deeper comprehension of the relationship between intracellular iron pools and magnetosome content at a single-cell level but also enables the investigation of the effects of varying iron and oxygen concentrations on cellular growth, magnetosome synthesis, and the formation of polyhydroxyalkanoate (PHA) granules.

## 2 Materials and Methods

### 2.1 Strains, Growth Media, and Culture Conditions

*Magnetospirillum gryphiswaldense* MSR-1 (DMSZ 6631) was grown in flask standard medium (FSM), comprising 3.5 g L^–1^ potassium L-Lactate, 0.1 g L^–1^ KH_2_PO_4_, 0.15 g L^–1^ MgSO_4_·7H_2_O, 2.38 g L^–1^ HEPES, 0.34 g L^–1^ NaNO_3_, 0.1 g L^–1^ yeast extract, 3 g L^–1^ soy bean peptone, 5 mL L^–1^ EDTA-chelated trace elements solution (EDTA-TES), and 0–1 mM iron citrate (C_6_H_5_FeO_7_). For all experiments, an iron-free modified version of EDTA-TES solution was employed, consisting of 5.2 g L^–1^ EDTA disodium salt; 30 mg L^–1^ H_3_BO_3_; 85.4 mg L^–1^ MnSO_4_·H_2_O; 190 mg L^–1^; CoCl_2_ g L^–1^; 4 mg L^–1^ NiCl_2_·6H_2_O; 2 mg L^–1^ CuCl_2_·2H_2_O; 44 mg L^–1^ ZnSO_4_·7H_2_O, and 36 mg L^–1^ Na_2_MoO_4_·2H_2_O. The pH of FSM and EDTA-TES was adjusted to 7.0 and 6.5, respectively, with NaOH prior to autoclaving.

For the first experiment, aerobic and microaerobic MSR-1 cultures were grown under different iron citrate dosages (0–50–100–300 μM) to compare the effects caused by these environmental factors on magnetosome formation and the intracellular iron pool. An additional experiment investigating MSR-1 microaerobic growth under a broader range of iron citrate dosages (0–100–300–500–1000 μM) was conducted to further explore iron tolerance. Both experiments were performed in hungate-type tubes (16 × 125 mm, Chemglass Life Sciences) with working volumes of 10 mL. Each tube was inoculated at a 1:10 ratio, and samples were collected before the end of the exponential growth phase (approximately at 72 h).

For the time-course experiment in which the evolution of the intracellular iron pool and magnetosome formation was monitored over time, 100 mL DURAN bottles with working volumes of 40 mL were used. As the objective was to study the evolution of magnetosome formation over time, nonmagnetic MSR-1 cells were used to inoculate aerobic and microaerobic cultures supplemented with 100 μM iron citrate at an initial OD_565_ of 0.1. Samples were then collected at various time points (0, 4, 8, 24, and 28 h).

All MSR-1 cultures were grown in triplicate at 30 °C in an Incu-Shake MAXI (SciQuip Ltd., Newtown, UK) orbital shaker incubator operated at 150 rpm. To create microaerobic conditions, both bottles and hungate tubes were purged with N_2_ for 10–30 min to remove all of the dissolved O_2_ and sealed with bromobutyl rubber stoppers. Subsequently, the necessary volume of sterile air was injected to achieve an oxygen concentration of 1%.

### 2.2 Bacterial Growth and Magnetic Cellular Response

Bacterial growth was determined by measuring the optical density of cultures in an Evolution 300 UV–vis spectrophotometer (Thermo Fisher Scientific, Hemel Hempstead, Herts, UK) at a wavelength of 565 nm (OD_565_). Cellular magnetic response (*C*_mag_) was measured as described elsewhere immediately after obtaining the OD_565_ values.^23^ Briefly, the spectrophotometer was equipped with two pairs of Helmholtz coils positioned around the cuvette holder: one pair perpendicular to the light beam and the other one parallel. The OD_565_ values were measured under each condition. In the presence of magnetic cells, the alignment with the two orientations leads to different optical densities, while nonmagnetic cells will not be affected by the switch of the magnetic field, resulting in no change in optical density. *C*_mag_ values were calculated by dividing the OD_565_ values for cells aligned parallel and perpendicular to the light beam. *C*_mag_ values range from 1 to 3, with a value greater than 1 indicating the presence of magnetic cells.

### 2.3 Flow Cytometry

Bacterial samples were collected from the liquid cultures, diluted in phosphate-buffered saline solution (PBS), and directly analyzed in a BD Accuri C6 flow cytometer (Becton, Dickinson and Company, Oxford, UK). FCM was used to determine relative cell size (FSC-A), cell granularity/complexity (SSC-A), intracellular iron concentration, and PHA formation. The intracellular iron concentration was detected using the Phen Green SK fluorophore (PG-SK), and PHA granules were stained with Pyrromethene-546 (Pyr-546). Details of the fluorescent probe staining conditions are provided elsewhere.^23^ Fluorescent labeled cells were excited using a 488 nm solid-state laser, and fluorescence was detected using a 533/30 BP filter (FL1-A).

### 2.4 Determination of Iron Content

The intracellular and extracellular iron concentrations of MSR-1 cultures were analyzed using an ICP-OES (Thermo Scientific iCAP 7000) as an offline analysis. The iron concentration was determined at a wavelength of 259.94 nm. One mL of the sample was collected and centrifuged to separate cells from the culture media. The supernatant was acidified by adding 10 μL of 70% (v/v) nitric acid prior to analysis. Cell pellets were first washed using PBS to remove iron traces from the media and subsequently digested with nitric acid (70% v/v) at 98 °C for 2h with shaking at 300 rpm in a Thermo Mixer HC (Starlab, Blakelands, UK) prior to analysis. All values obtained were then normalized using the dry cell weight (DCW) to avoid discrepancies due to different cell biomass.

### 2.5 Transmission Electron Microscopy

Transmission electron microscopy images of magnetic MSR-1 cells grown under various iron dosages (0–100–300–500–1000 μM iron citrate) were captured using a JEOL 2100F FEG microscope (JEOL, Herts, UK) operated at 200 kV and equipped with a Gatan K3 IS camera. MSR-1 cells (2 μL) were deposited on a lacey carbon 300 mesh copper supported grid (GOLC300Cu50, EM Resolutions, Sheffield, UK) and vacuum-dried before analysis. To determine the mean length of the magnetosome chains, 100 cells were randomly selected for each condition, and the number of magnetosome crystals per chain was counted. Statistical analysis was conducted using one-way analyses of variance (ANOVA) followed by Bonferroni’s post-test to compare the effect of the different iron dosages on the magnetosome chain length. The cutoff value for statistical significance was set at *p* < 0.01.

### 2.6 CryoSIM and CryoSXT Correlative Microscopy Collection and Analysis

Vitrification of samples was done at the Diamond Light Source (DLS). Grids (QUANTIFOIL R 2/2 Au G200F1) were glow discharged before sample deposition using a PELCO easiGlowTM system. Before plunge freezing, samples were incubated with either PG-SK (1 mM – 5 min incubation) or Pyr-546 (0.1 mg mL^–1^) at room temperature. The grid was then mounted in the plunging apparatus (Leica EM GP) and 2–4 μL of sample was deposited on top of the carbon surface of the grid. Before fiducial deposition, manual blotting from behind was performed using Whatman filter paper. Once fiducials were deposited (2 μL of either 150 or 250 nm gold nanoparticles), the sample was automatically blotted from behind (on one side) for 0.5–1 s at room temperature in an environmentally controlled chamber at 70% humidity. Vitrification was achieved by rapidly plunging the grid into liquid ethane maintained at −170 °C. Finally, the grids were stored in liquid nitrogen until further analysis. Three replicates were prepared for each experimental condition.

Vitrified samples were loaded in a Linkam cryostage coupled to the cryoSIM microscope at beamline B24 for SIM data collection.^29^ To identify regions of interest (ROIs), each grid was mapped by acquiring 2D bright-field transmission mosaics. Once ROIs were annotated, bright-field and SIM 3D data were collected along the *z*-axis. PG-SK and Pyr-546 fluorescence data were collected using a green laser (488 nm wavelength) for 40 ms (exposure time) at 50 mW of power. Fluorescence was collected through a 525 nm filter on a CCD camera.

For cryoSXT data collection, the vitrified samples were transferred to the UltraXRM-S/L220c transmission soft X-ray microscope (Carl Zeiss) at beamline B24. Grids were first visualized using an inline visible-light 20x objective to identify previously annotated ROIs. For cryoSXT collection, the transmitted light was focused using a 25 nm zone plate (X-ray objective), resulting in a 10 μm × 10 μm field of view. 2D X-ray mosaics of ROIs were captured to identify interesting areas for tomography collection. Tilt series were collected using an incident beam of 500 eV (“Water Window”) or 710 eV (Fe L_3_-edge) at tilt angles from −60° to +60° with a step size of 0.5° and an exposure time of 0.5 s per frame.

After tilt series were acquired, tomograms were reconstructed either manually using IMOD^©^ (University of Colorado)^32^ or by an automated pipeline based on IMOD (batchruntomo) using a simultaneous iterative reconstruction technique. To correlate both cryoSTX and cryoSIM data sets, eC-CLEM software was employed.^33,34^ To measure individual cell PG-SK fluorescence intensity, Fiji (ImageJ) software was used. SuRVos2 Workbench^35^ was used to segment cellular features such as the magnetosome chain, PHA granules or the cellular membrane and the 3D volume render was visualized using UCSF ChimeraX.^36^

## 3 Results and Discussion

### 3.1 Effect of Iron and Oxygen Availability on MSR-1 Growth and Magnetosome Biogenesis

Given the key roles that iron and oxygen play in magnetosome biomineralization,^37^ MSR-1 was grown under different oxygen and iron conditions. Samples were then collected at the end of the exponential growth phase for each condition and analyzed.

**Figure 1:**
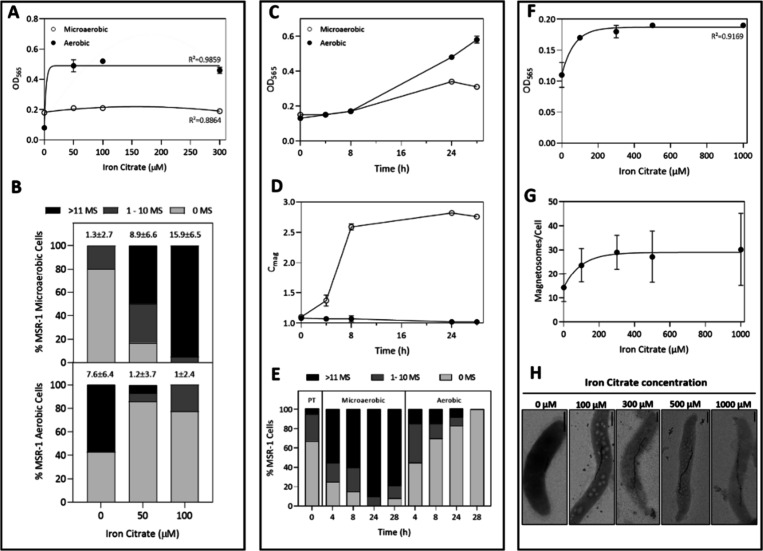
Effect of iron and oxygen availability on MSR-1 cell growth and magnetosome synthesis. (A, B) MSR-1 cells were grown under different iron dosages (0–50–100–300 μM iron citrate) under either microaerobic or aerobic conditions. (C-E) Time-course experiment in which MSR-1 cells were grown at 100 μM iron citrate under microaerobic or aerobic conditions over a period of 28h. (F-H) Iron tolerance test of MSR-1 cells grown under microaerobic conditions. The iron dosage range for this experiment was 0–100–300–500–1000 μM iron citrate. (A-F) Cell growth was monitored by measuring the optical density of the different cultures (OD_565_). (D) Cellular magnetic response (*C*_mag_) values obtained from time-course experiment. (B, E) Representation of magnetosome content of the two main correlative microscopy experiments (n_(B)_= 102; n_(E)_= 204). For each condition, magnetosome content was classified according to the magnetosome chain length: long chain (>11 MS), short chain (1–10 MS) or no chain (0 MS). (G) Mean magnetosome chain length of iron tolerance test (*n* = 500). (H) TEM images of iron tolerance test of MSR-1 cells. Error bars are standard deviation. MS = magnetosomes; PT = pretransfer; scale bar = 500 nm.

The effect of oxygen on MSR-1 growth is depicted in Figures 1A and 1C. As can be observed, there is a growth reduction in microaerobic (low oxygen) conditions compared to aerobic conditions. This observation is in line with previously reported data.^37^ In all known MTB, when oxygen is abundant, magnetite formation is suppressed, allowing the bacteria to allocate more energy toward cell growth and aerobic respiration.^4^ For instance, it has been demonstrated that genes related to nutrient transport and physiological metabolism are down-regulated under microaerobic conditions as energy is redirected for magnetosome synthesis.^38^

As for the effect of iron concentration on cell growth, several observations were noted. First, the absence of iron in the media hinders cell growth, as evidenced in Figures 1A and 1F.^39^ A significant decrease in the cellular growth of MTB is not surprising given that iron is a key player in many biological reactions and metabolic pathways.^1^ Importantly, aerobic cell growth was shown to be more significantly affected by the absence of iron than microaerobic cultures, given that a complete lack of growth was observed under this condition. This trend has been consistently observed when an EDTA-TES iron-free solution was utilized for aerobic cultures in preliminary experiments (data not presented). Second, the increase of iron citrate dosage did not exhibit any detrimental impact on cellular growth up to 300 μM (Figure 1A). Despite literature suggesting that iron toxicity for MSR-1 is set at concentrations exceeding 200 μM,^39,40^ such effects were not observed in our experimental set up. This discrepancy may be due to differences in experimental methodology, particularly, the buffering capacity of the media. Consequently, a further experiment was conducted to assess the MSR-1 iron tolerance. The results of this complementary experiment (Figure 1F) showed that cell growth remained unaffected even with an increase of iron concentration up to 1 mM. This observation, along with previous findings in MSR-1 fermentation experiments,^4,41^ supports our hypothesis that MSR-1 endures greater tolerance to high iron concentrations than initially presumed. This suggests the existence of robust iron regulatory mechanisms that remain poorly understood, such as iron deposition into bacterioferritins^14^ or the molecular mechanisms involved in avoiding reactive oxygen species (ROS) accumulation.^42^

The number of magnetosomes per cell for each iron and oxygen condition was determined by using cryoSXT (Figure 1B). Due to the resolution limitations of this technique, only magnetosomes containing magnetite crystals, and not empty magnetosome vesicles, can be detected. Therefore, from this point forward, any reference to the number of magnetosomes pertains exclusively to those containing a magnetite crystal. It should be noted that, unfortunately, grids corresponding to the 300 μM conditions have not be screened by cryoSXT due to time constraints. Magnetosome chains from all other conditions successfully screened by cryoSXT were then classified as short or long, depending on whether they contain less than or more than 10 magnetite crystals, respectively. Based on our analysis, microaerobic cells grown under a low iron concentration (50 μM) exhibited shorter chains and a more heterogeneous chain length distribution, as only 50% were considered long. In contrast, 95% of cells grown at a higher iron dosage (100 μM) presented long chains (Movie S1), reflecting a more homogeneous chain length distribution. These results suggest that higher extracellular iron concentrations may lead to the production of longer magnetosome chains. Nonetheless, iron tolerance test results (Figure 1G) showed that for iron citrate concentrations higher than 300 μM, magnetosome chains were not significantly longer (one-way ANOVA with Bonferroni test, *p < 0*.01). Other studies have reported that magnetosome yields do not improve when using iron citrate concentrations higher than 200 μM.^40^ Around 80% of microaerobic cells grown without iron did not contain any magnetosome chain. However, some cells still had short magnetosome chains because of the presence of trace amounts of iron in the media carried over from precultures (Figure 1H and Movie S4).

Although it is widely known that magnetite formation is suppressed under aerobic conditions,^37,39^ some magnetosome production can still be observed under our experimental conditions. Since iron-starved aerobic cells did not grow, the amount of magnetosomes per cell (reflected in Figure 1B) corresponds to the initial presence of magnetic cells in the starting culture for this experiment. The presence of magnetite crystals under the 50 and 100 μM aerobic conditions can be attributed to the air transfer restriction caused by the use of sealed tubes with only 38% of headspace. As cell growth progressed, oxygen contained in the tubes was consumed, prompting cells to switch to microaerobic metabolism and activate magnetosome production. This increase in magnetosome formation due to oxygen depletion has been observed at both flask and bioreactor scales either by limiting the flask headspace volume^23^ or ceasing bioreactor aeration.^4,5^ In line with recent findings by Pang et al., oxygen depletion triggers denitrification pathways that allow for the generation of nitric oxide (NO), which is crucial for the expression of biomineralization genes. Under aerobic conditions, oxygen respiration may compete with nitrification/denitrification pathways and thereby impairing magnetite formation.^43^

Due to the complexity of controlling oxygen limitation in tubes, a separate experiment was performed using 100 mL bottles where the headspace was larger (70%) and aerobic conditions could be prolonged over time. In this experiment, MSR-1 cells were grown using 100 μM iron citrate under either aerobic or microaerobic conditions. Then, parameters such as cell growth (Figure 1C), magnetic cellular response (*C*_mag_) (Figure 1D) and magnetosome content (Figure 1E) were monitored over time. To facilitate the monitoring of magnetosome biomineralization from its onset, a low-magnetic preculture was used (*C*_mag_ = 1.1). Both *C*_mag_ values (Figure 1D) and magnetosome content (Figure 1E) clearly show that magnetosome production increased for cells under microaerobic conditions and reduced to *C*_mag_ ∼ 1 for cells exposed to aerobic conditions. The peak of magnetosome production in microaerobic cultures occurred at the end of the exponential phase as previously observed by others.^4,5^ Magnetosome content in aerobic cells was roughly halved at every sampling time point. During MSR-1 cell division, the magnetosome chain is cleaved into two and each fragment passed onto a daughter cell.^44^ Therefore, the magnetosome chains present in the magnetic cells of the starting culture did not disappear immediately after continuous exposure to air. As cells divide, the chains are split and become shorter until cells are depleted of magnetosomes.

### 3.2 Effect of Iron and Oxygen Availability on the Intracellular Iron Pool

Up until recently, it was thought that magnetosomes constituted 99% of the intracellular iron content.^11^ However, recent studies indicate that only 25–45% of the intracellular iron is bound in magnetite.^18,19^ The remaining fraction is used in other general iron-dependent biochemical reactions^7,21^ or deposited in iron-storing proteins such as bacterioferritins to avoid a toxicity effect.^14^ The existence of these different intracellular iron pools adds more complexity and difficulty in elucidating the relationship between the intracellular iron pool and magnetosome formation. To this end, an innovative multipronged approach involving different techniques was combined to obtain a holistic view of the effects of the extracellular iron concentration of magnetic and nonmagnetic MSR-1 cells on the intracellular iron pool and how this correlates with magnetosome formation.

Quantification of the total amount of intracellular iron was done by ICP-OES, whereas FCM was used to assess the labile Fe^2+^ intracellular pool using a fluorophore probe called PhenGreen SK (PG-SK). PG-SK is a chelatable fluorophore that has high affinity for iron and is quenched when it binds to it. PG-SK has been a useful tool for the detection of the intracellular iron pool in MSR-1,^22,23^ as well as in other eukaryotes^45^ and bacteria.^46^

**Figure 2:**
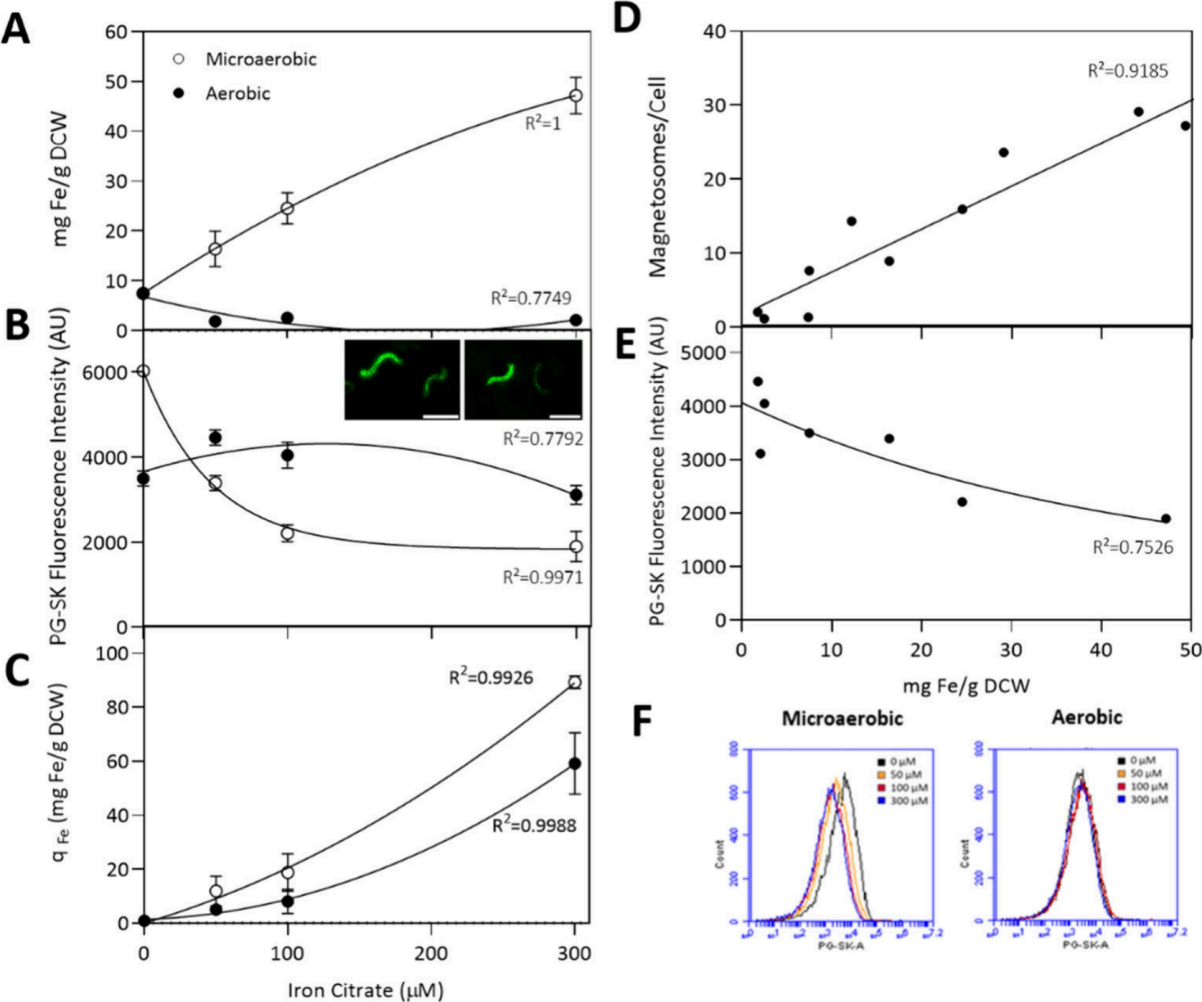
Effects of iron concentration and oxygen availability on the intracellular iron pool of MSR-1 cells grown under different iron dosages (0–50–100–300 μM iron citrate) under microaerobic and aerobic conditions. (A) Total intracellular iron concentration normalized to biomass (DCW = dry cell weight). Error bars represent standard deviation. (B) PG-SK fluorescence intensity values and (F) fluorescence histograms obtained from FCM (flow cytometry) analysis. 25,000 events were analyzed per sample by FCM; error bars show covariance. Insets images correspond to CryoSIM images of MSR-1 cells stained with PG-SK. Scale bar = 2 μm. (C) q_Fe_ iron uptake rates. (D) Correlation between the total intracellular iron concentration and magnetosome units per cell. (E) Correlation between the total intracellular iron concentration and PG-SK fluorescence intensity obtained from FCM. AU = arbitrary units.

The quantitative estimation of the total amount of iron in MSR-1 cells grown under microaerobic conditions (Figure 2A) revealed a clear correlation between the intracellular iron pool and the supplemented iron from the growth media. This indicates that an increase in extracellular iron leads to a corresponding increase in the intracellular iron concentration. However, we have found that for conditions in which the initial iron levels in the media exceeded 300 μM, a plateau was reached in which the intracellular iron concentrations remained unchanged even with further increases in iron supplementation (Figure S1). Similar results have also been observed for the AMB-1 strain grown under different iron dosages.^24^ Notably, our measurements of *q*_Fe_ iron uptake rates (Figure 2C) demonstrates a correlation with the extracellular iron concentration, indicating that MSR-1 enhances its iron uptake capacity as more iron becomes available in the environment, most likely by active transport mechanisms such as ABC or FeoB membrane proteins.^47^ Amor et al. reported that iron incorporation in the phylogenetically close strain AMB-1 was 10-fold higher under high iron conditions (150 μM) compared to low iron conditions (30 μM).^19^ In contrast, under aerobic conditions, intracellular iron concentrations were notably lower compared with those observed under microaerobic conditions and remained stable despite increases in the initial iron concentration in the media (Figure 2A). As mentioned in the previous section, the depletion of oxygen in the tube headspace leads to a metabolic shift from aerobic to microaerobic metabolism. This explains why we observed a similar trend in aerobic *q*_Fe_ iron uptake rates but with lower values and a gentler fitting slope.

Additionally, a clear linear correlation was observed between the magnetosome content and intracellular iron concentration (Figure 2D). Specifically, as the length of magnetosome chains increased, there was a corresponding increase in the concentration of the total intracellular iron.

Upon examination of the intracellular labile Fe^2+^ pool using FCM, aerobic PG-SK fluorescence values were higher than in microaerobic conditions (Figure 2B and Figure S2). Considering that PG-SK quenches upon Fe^2+^ bonding, the decrease of green fluorescence is proportional to the size of the labile Fe^2+^ pool. Similar to the findings observed in the total iron concentration (Figure 2A), an indirect positive correlation was found in microaerobic conditions between the initial iron dosage in the media and the corresponding intracellular Fe^2+^ concentrations, in which the increase of the initial iron dosages caused the increase of the intracellular Fe^2+^ levels. Despite ferric (Fe^3+^) iron (iron citrate) being the only source of iron provided in the media, it has been proven that MSR-1 employs ferric reductases to convert Fe^3+^ to Fe^2+^^12,13^ and uses general iron uptake systems such as the Feo system to incorporate ferrous iron.^8,9^ Gene expression studies during cell growth and magnetosome synthesis revealed that under high-iron conditions, ferric reductase genes, ferrous transport system-related genes, and ROS scavenging related genes are highly expressed whereas under poor iron conditions only iron transport-related genes are expressed.^48^ This enhanced expression of ROS scavenging-related genes might be the key to understanding how MTB can take up such high amounts of iron or live within these excessive iron concentrations.

Comparison of ICP-OES and FCM data reveals an inverse correlation between the total iron concentration and PG-SK fluorescence values (Figure 2E). Due to the quenching nature of PG-SK upon iron binding, this inverse correlation implies an actual direct relationship with the Fe^2+^ concentration. Fluorescence intensity histograms (Figure 2F) also reflected the distinct variations in PG-SK fluorescence in microaerobic conditions with respect to the nonvariant fluorescence profiles of the aerobic conditions. All conditions displayed a unimodal distribution but with wide peaks. This indicated that the fluorescence was not uniform among cells grown under the same conditions. This heterogeneity in cellular fluorescence was confirmed by fluorescence microscopy observations (Figure 2B insets). FCM population analysis (Figure S3) indicated that this variability in fluorescence could be attributed to the presence of two distinct cell populations. The first population was comprised of smaller cells with elevated intracellular Fe^2+^ levels, while the second consisted of larger cells with diminished intracellular iron concentrations. This pattern was consistent across all of the experimental growth conditions.

Based on the findings, cells with higher intracellular labile Fe^2+^ concentrations are likely to possess a higher number of magnetosomes compared with those with lower iron levels. Despite the evidence provided by ICP-OES and FCM data, this hypothesis could not be verified. Therefore, to corroborate this hypothesis, we performed for the first time a single-cell analysis using a synchrotron-based correlative microscopy approach involving cryoSXT and cryoSIM.^29^ CryoSXT enabled direct visualization and characterization of magnetite formation, while cryoSIM was employed to indirectly examine the intracellular distribution of iron by locating its chemical signals in PG-SK-stained cells.

**Figure 3:**
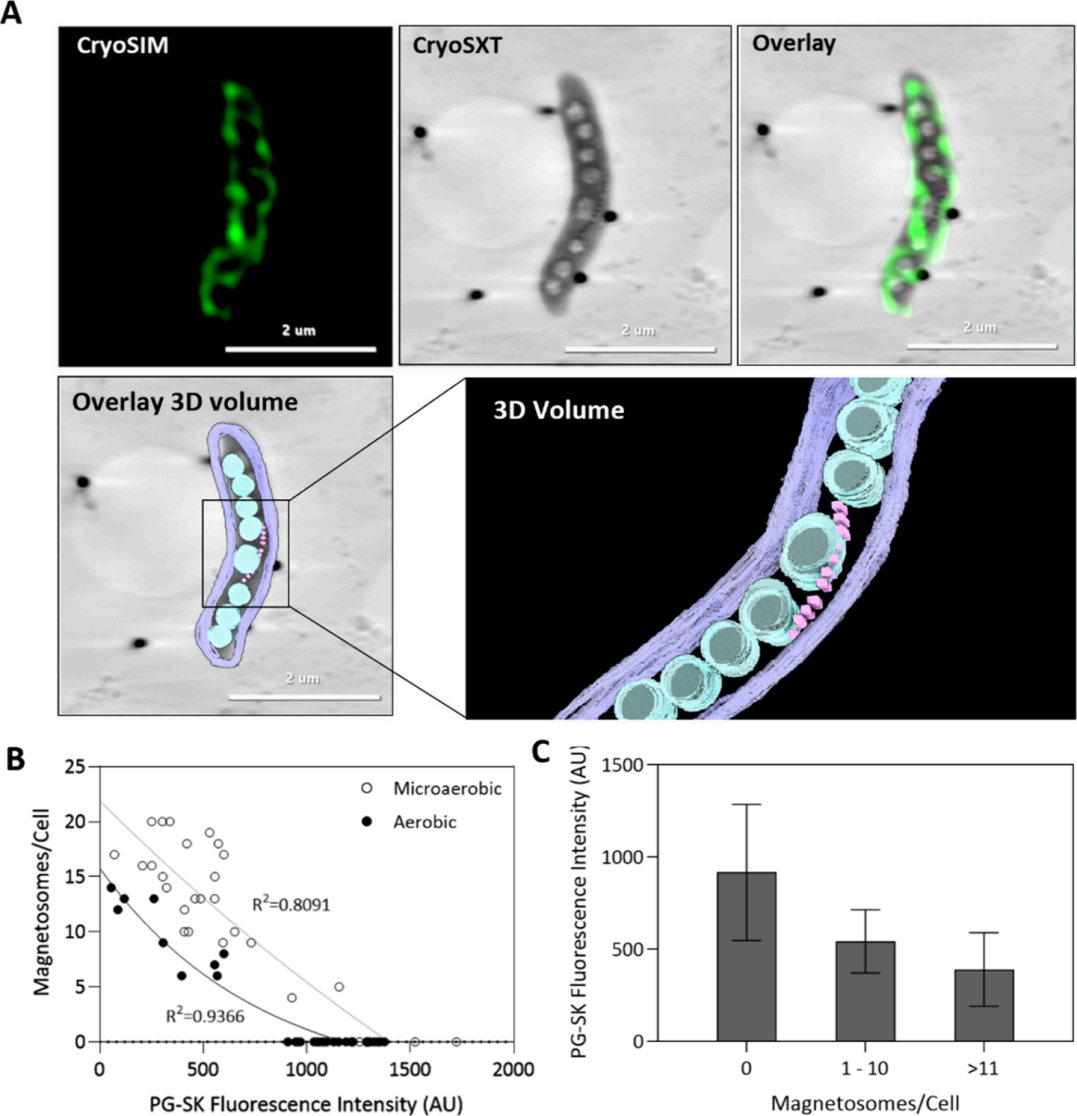
(A) Representative tomogram slices of the MSR-1 magnetosome producing cell grown under microaerobic conditions imaged by cryoSIM and cryoSXT, including a 3D volumetric representation of this cell using a SuRVos2 workbench. Magnetosomes are colored in pink, PHA granules in blue and the cell membrane in purple. (B) Single-cell correlation between cryoSIM PG-SK fluorescence and number of magnetosome crystals per cell of microaerobic and aerobic MSR-1 cells (*n* = 63). (C) Mean cryoSIM PG-SK fluorescence values of MSR-1 cells with no magnetosomes, short magnetosomes chains (1–10) and long magnetosome chains (>11) (*n* = 104). AU = arbitrary units.

MSR-1 cells grown under varying iron and oxygen regimes were vitrified and screened by cryoSXT and cryoSIM at beamline B24 at the UK synchrotron, Diamond Light Source. Within the soft X-rays spectrum (0.1–1 keV), it is feasible to distinguish elements based on their absorption properties. There is a particular spectral region known as the “water window” located between the absorption edges of carbon (284 eV) and oxygen (543 eV) that is especially advantageous for visualizing biological specimens because carbon- and nitrogen-rich components absorb X-rays strongly, compared to the oxygen-rich media that surround them, without the need to add any contrast staining reagents.^49^ X-ray tomographs of MSR-1 imaged at the “water window” (500–510 eV) were acquired alongside 3D cryoSIM data from the same cells (Figure 3A and Movie S2). PG-SK green fluorescence was homogeneously distributed in the cytoplasm except in regions containing PHA granules that remained unstained. For each individual cell, the number of magnetosomes was counted, and PG-SK fluorescence intensity was calculated using the Fiji imaging software. The corresponding single-cell correlation data is shown in Figure 3B. Cells with a higher count of magnetosomes displayed lower levels of PG-SK fluorescence, indicating elevated intracellular ferrous iron concentrations. Inversely, a decrease in the number of magnetosomes per cell caused a reduction in the intracellular Fe^2+^ pool under both microaerobic and aerobic conditions. However, it is important to note that in the aerobic condition cells likely entered a microaerobic state during growth due to oxygen consumption and limited oxygen exchange in the hungate tubes. This transition may have influenced the intracellular iron concentrations observed in the aerobic samples. The number of magnetosomes per cell and their correlation with PG-SK fluorescence values was consistent whether PG-SK fluorescence was obtained via flow cytometry (Figure S4) or from cryoSIM data (Figure 3B), as similar trends were observed.

Moreover, when cells were analyzed solely based on the length of the magnetosome chain, regardless of the different growth conditions, significant differences were observed (one-way ANOVA with Bonferroni test, *p* < 0.01) with regards to the PG-SK fluorescence intensities among cells classed as “no” (zero), “long” (>11), or “short” (1–10) magnetosome chain length (Figure 3C). Cells with long magnetosome chains exhibited the highest intracellular Fe^2+^ iron concentrations, while those without presented the lowest Fe^2+^ levels. Notably, 85% of cells with long magnetosome chains were grown under microaerobic conditions, whereas 65% of magnetosome-lacking cells were grown aerobically (Figure S5). Cells with short magnetosome chains had a more balanced distribution between cells grown under restricted oxygen (56%) and cells grown under aerobic conditions (44%). To the best of our knowledge, this level of analyses has not been performed before this work; hence, we provide new insights into the elucidation of iron uptake dynamics and its correlation with magnetosome formation in MSR-1.

### 3.3 Evolution of the Intracellular Iron Pool during Biomineralization

In the previous section, the focus was on the impact of iron and oxygen availability on the intracellular iron pool and magnetosome formation. MSR-1 cells were harvested at the end of their exponential growth phase as magnetosome chain length is typically at its peak in their bacterial cell cycle.^5^ To further explore the relationship between the intracellular iron pool and magnetosome formation, we conducted a time-course study aiming to monitor the changes in the intracellular iron pool as magnetosome chains form in time. MSR-1 cells were grown under identical initial iron citrate conditions (100 μM) under microaerobic and aerobic conditions. Samples were subsequently taken at several time points and analyzed using the same analytical and correlative microscopy techniques employed in the preceding experiment.

**Figure 4:**
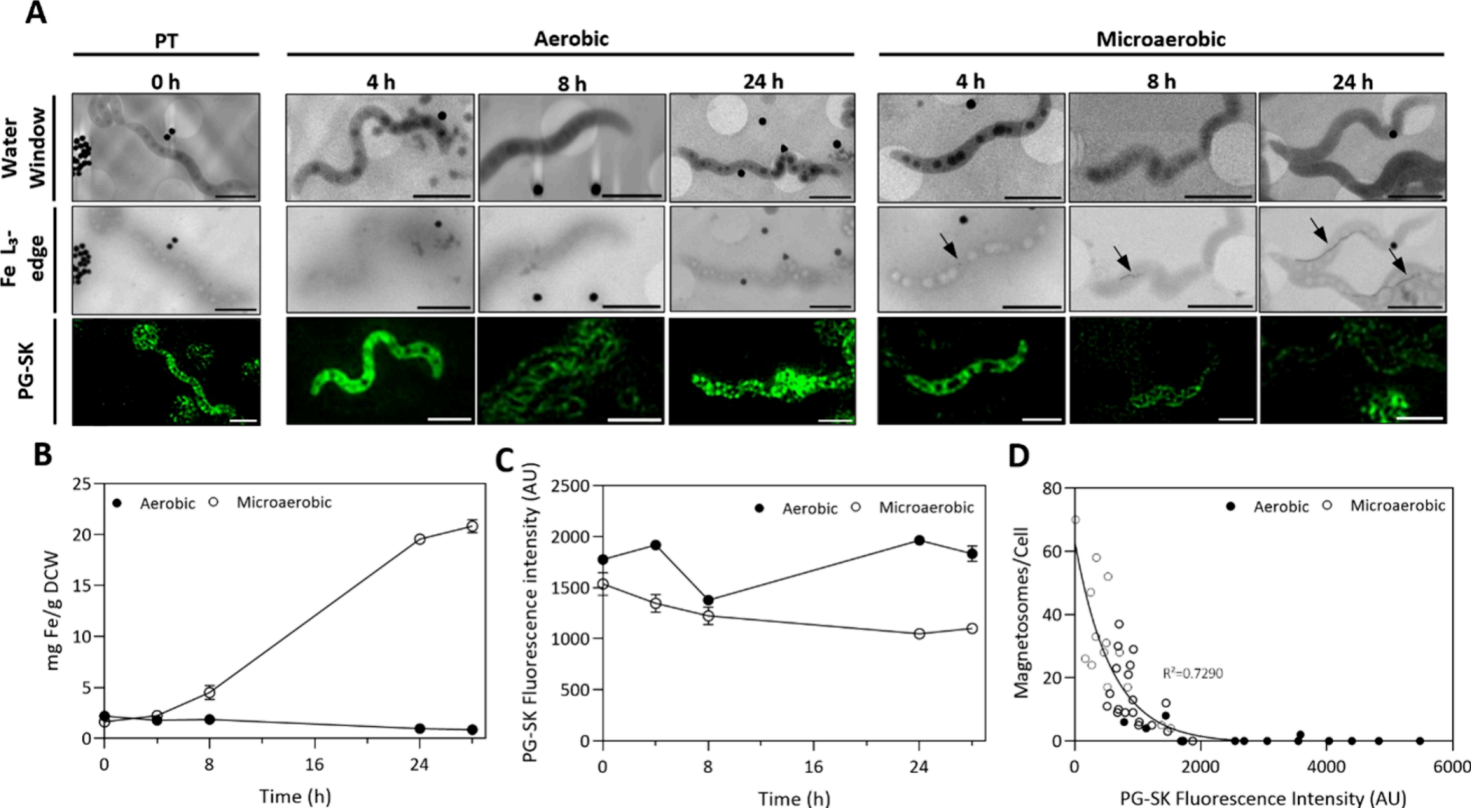
(A) Representation of magnetosome biomineralization evolution illustrated by cryoSXT and cryoSIM images of MSR-1 cells grown under microaerobic or aerobic conditions over a period of 24 h. Two different photonic energies were employed for cryoSXT acquisition: 500 eV (top row) and 710 eV (middle row). MSR-1 cells were stained with a PG-SK fluorescent probe before the cryoSIM data acquisition. Arrows indicate the presence of the magnetosome chains. Evolution of (B) the total intracellular iron concentrations and (C) PG-SK mean fluorescence intensity values (FCM) of MSR-1 cells grown with 100 μM iron citrate under microaerobic or aerobic conditions. (D) Single-cell correlation between PG-SK fluorescence (cryoSIM) and the number of magnetosome crystals per cell of microaerobic and aerobic MSR-1 cells grown under 100 μM iron citrate (*n* = 62). Error bars indicate the standard deviation (*n* = 3). Scale bar = 2 μm. PT = Pretransfer. AU= arbitrary units.

Figure 4A illustrates the magnetosome biomineralization process over time. Visual inspection of the cryoSIM images (bottom row) revealed a striking decrease in PG-SK fluorescence as magnetosome chains elongated under microaerobic conditions. It was also observed that some of the cells containing the longest chains barely exhibited any fluorescence, indicating substantial accumulation of Fe^2+^. Contrary to what Amor et al. observed when using another ferrous fluorescent probe (FIP-1),^19^ we did not observe localized fluorescence. They reported Fe^2+^ accumulation around the magnetosome chain and at the cell poles during cell division. However, due to the quenching properties of the PG-SK fluorophore, we were unable to quantify any localization. Our observations were limited to the turn-off nature of the fluorophore when quenched by Fe^2+^.

One advantage of using soft X-rays for imaging cellular structures is the ability to visualize different elements depending on the chosen X-ray energy within the spectrum.^50^ In this experiment, in addition to imaging at an energy within the “water window” (Figure 4A – top row), where water is transparent and carbon absorbs X-rays heavily thus generating contrast, we also imaged our samples by changing the energy of the transmission X-ray microscope at beamline B24 to the Fe L_3_ absorption edge (Figure 4A – middle row). Magnetosomes presented higher contrast at the Fe L_3_ absorption edge (ca. 710 eV), making them more discernible compared to the “water window” (Movie S3 versus Movie S4), as iron-rich elements selectively absorbed X-rays at this energy.^51^ Additionally, microaerobic cells appeared darker overtime at the Fe L_3_ edge (Figure 4A – middle row), indicating an increase in cellular iron content during magnetosome formation. This increased dark contrast due to iron accumulation in the cytoplasm also enhanced the visibility of cell structures such as PHA granules or cell membranes by the end of the biomineralization process. In contrast, as aerobic cells exhibited low intracellular iron content, the cytoplasm had less contrast, and cells appeared more blurred, which challenged the visualization of cell structural details compared to microaerobic cells.

Total intracellular iron concentration results (Figure 4B) determined by ICP-OES confirmed the variance in iron content observed between microaerobic and aerobic conditions already evidenced in the L_3_-Fe edge data. Results shown in Figure 4B are consistent with the C_mag_ results (Figure 1D). In both graphs, it is apparent that the start of iron accumulation occurred at the beginning of the exponential growth phase (*t* = 8 h), a period in which magnetosome production started to be significant, while the iron concentration and *C*_mag_ values under aerobic conditions remained consistently low throughout the duration of the experiment as biomineralization is inhibited in the presence of oxygen.^37^

Time-course PG-SK fluorescence intensity results obtained by FCM (Figure 4C) were consistent with those obtained from cryoSIM fluorescence measurements (Figure S6). Microaerobic cells exhibited a 1.5-fold decrease in green fluorescence, indicating an increase in Fe^2+^. This modest increase in labile Fe^2+^ concentration compared to the 9-fold increase observed in the total amount of intracellular iron (Figure 4B) suggests that labile Fe^2+^ is not the only form of iron present at the end of the biomineralization process, distinct from magnetite. As reported by Amor et al. and Berny et al. in separate studies, only 25–45% of the total intracellular iron corresponds to magnetite^18,19^ and this study supports this hypothesis. The other suggested iron species constituting the intracellular iron pool, aside from magnetite, are likely to be associated with proteins that contain heme-binding domains^52^ or iron storage proteins, such as bacterioferritins.^7,14^ Nevertheless, further analytical and spectroscopic studies (e.g., scanning transmission X-ray microscopy) are required to identify the presence of these compounds during biomineralization to verify this hypothesis. The identification of all iron-uptake and regulation mechanisms involved in magnetosome synthesis can also enhance our comprehension of the composition of the intracellular iron pool and the specific roles that each component plays in the biomineralization process. Evidence indicates that general iron uptake systems participate in transporting iron into the intracellular space.^53^ However, it has been suggested that an additional iron uptake pathway specific to biomineralization may exist. The variability of different general iron uptake mechanisms and homeostasis systems among MTB species supports this hypothesis. For instance, siderophore synthesis has been reported for AMB-1, MV-1, and MS-1 strains, but not for MSR-1.^54−56^ To further support this hypothesis, suppression of FeoB1 ferrous iron transporter^8^ or ferritin expression in MSR-1^14,57^ led to reduced, but not completely suppressed, biomineralization, suggesting the existence of additional, yet unidentified, iron uptake mechanisms.

Figure 4D shows the correlation between the number of magnetosomes per cell and the PG-SK intensity values at the single-cell level. This correlation was comparable to that observed between magnetosomes per cell and PG-SK intensity values obtained by using FCM (Figure S7). Additionally, the relationship between magnetosome content and PG-SK fluorescence is consistent with the one observed in the previous correlative microscopy experiment shown in Figure 3B. Cells with a greater number of magnetosomes per cell displayed lower green fluorescence, indicating higher concentrations of Fe^2+^ compared to the cells with fewer magnetosomes. However, it is worth noting that other nonproducing magnetosome cells followed a different correlation pattern. Such cells displayed the highest fluorescence values, but as shown in Figure 4D, some of these cells exhibited three times more fluorescence than other nonproducing magnetosome cells. This observed increase in fluorescence only occurs from 24 h onward and it further highlights the variability in fluorescence among the cell population.^4,23^

Among the various models proposed for MTB iron uptake, our results discarded the possibility of direct iron transport into the magnetosome vesicle lumen during membrane invagination, as no iron localization was detected in those areas prior to magnetite synthesis.^6^ Instead, our findings align with MTB iron uptake models suggesting that iron is first accumulated in the cytoplasm before being transported to the magnetosome vesicles.^7,8,21^ Earlier studies on magnetosome biomineralization primarily involved bulk spectroscopic characterization and assumed that the iron species, other than magnetite, that were detected during the early stages of magnetosome formation (e.g., ferritin, hematite) did not persist until the end of the biomineralization process.^7,15−17^ These analyses conducted using cells in bulk rather than single-cell analysis likely resulted in the magnetite signal overshadowing other iron species present in the cells, making it difficult to detect these additional signals. Hence, this emphasizes the significance and merit of employing single-cell analysis. Chevrier et al. also observed a significant presence of intracellular iron species different from magnetite in MSR-1 cells during biomineralization through single-cell analysis using nano-X-ray fluorescence mapping and nano-X-ray absorption near-edge structure analysis (nano-XANES).^20^ Consistent with our results, the authors also observed that the intracellular iron pool does not deplete after the synthesis of magnetosome chains. While nano-XANES is effective for chemical identification of iron species, its time-consuming nature limits the number of cells that can be analyzed, making it challenging to obtain statistically significant data. Additionally, the requirement to dry cells prior to analysis means that the observations may not fully represent their native conditions. In contrast, our study utilized cryopreservation to maintain samples in a fully hydrated near-native state, allowing for observations that more accurately reflect physiological conditions within MSR-1 cells. Furthermore, the efficiency of our approach enabled screening of a larger number of cells at the single-cell level. The use of a fluorophore specific for Fe^2+^ also provided qualitative iron speciation under near-native conditions, offering additional insights into the cellular processes.

### 3.4 Effect of Iron Dosage and Oxygen Regime on PHA Formation

It is widely known that PHAs are intracellular inclusions synthesized under conditions of carbon excess, nutrient limitation, or environmental stress.^58^ Although extensive research has been conducted on PHA synthesis in various microorganisms, studies focusing on PHA production in MTB remain limited. Here, the fluorescent probe Pyrromethene-546 (Pyr-546), which stains PHA granules green, was used to assess PHA content by FCM. Pyr-546 dye is a commonly used probe to detect PHA granules^59^ and has previously been used in studies involving MSR-1.^4,23^

**Figure 5:**
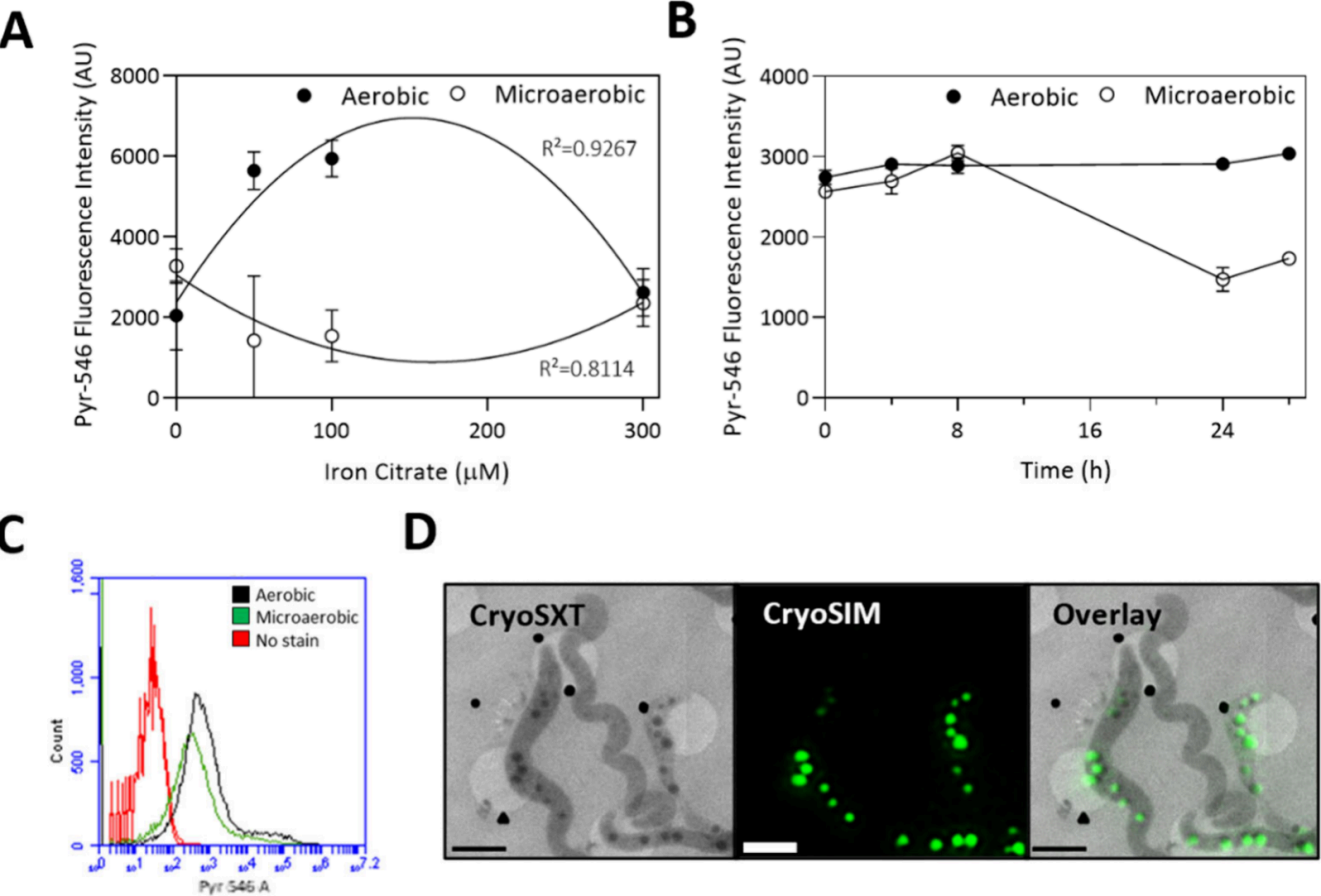
Analysis of the effect of iron and oxygen availability on PHA formation using FCM. (A) Comparison of Pyr-546 fluorescence values of MSR-1 cells grown under different iron dosages (0–50–100–300 μM iron citrate) under microaerobic or aerobic conditions. Errors bars indicate covariance. (B) Evolution of Pyr-546 fluorescence values between microaerobic or aerobic MSR-1 cells grown with 100 μM iron citrate over a period of 28 h. Error bars are standard deviation. (C) Comparison between fluorescence intensity histograms of microaerobic and aerobic MSR-1 cells stained with Pyr-546. (D) CryoSXT and cryoSIM images of MSR-1 cells stained with Pyr-546. Scale bar = 2 μm. AU = arbitrary units.

Our results indicate that aerobic conditions favor PHA formation in MSR-1 cells over microaerobic conditions, independent of the initial iron concentration in the media (Figure 5A and Figure 5B). Su et al. studied the effects of aerobic and anaerobic conditions on PHA production in AMB-1 strain and observed a reduction in PHA content when switching to anaerobic conditions, and an increase when switching to aerobic conditions.^60^ Notably, the absence of iron led to the highest PHA content under oxygen-limited conditions (Figure 5A and Figure S8), suggesting that iron limitation may induce stress responses that promote PHA synthesis as a storage mechanism. Previous work in the group also observed higher Pyr-546 fluorescence values in MSR-1 cells grown in the absence of iron compared to cells grown with iron.^23^ Interestingly, iron did not accumulate in or around PHA granules (Figure 4A – middle row) and the additional stress of being grown under high iron concentrations did not cause an increase in PHA content (Figure S8). These results suggest that oxygen may play a more important role in PHA synthesis and regulation than other stress factors such as a high iron concentration.

The evolution of PHA content over time when comparing aerobic and microaerobic conditions (Figure 5B) is highly relevant and enables us to understand the relationship between PHA and magnetosome synthesis. Previous studies have suggested the existence of an energy competition between magnetosome and PHA formation.^4,23,60,61^ When PHA synthesis was suppressed by genetic modification, magnetosome production was increased.^62^ Our results also reflect this competitive relationship. As shown in Figure 5B, PHA formation was reduced when significant magnetosome production started (*t* = 8 h), while under conditions inhibiting magnetosome synthesis (aerobic cultures), PHA content remained stable over time.

Pyr-546 fluorescence histograms (Figure 5C) reveal heterogeneity in the PHA content. A prominent peak is observed accompanied by a smaller peak or tail, which indicates the presence of a subpopulation with significantly higher PHA levels. Such variability, has been observed in other studies carried out with MSR-1^23^ as well as in other bacterial species.^63,64^Figure 5D shows evidence of this variability in the PHA content among cells grown under the same conditions. Karmann et al. suggested that these differences could be due to different cellular growth rates, distinct ability to degrade PHA or an asymmetric PHA distribution during cell division.^63^

FCM population analysis identified two distinct cell populations: smaller, simpler cells with higher intracellular Fe^2+^ and lower PHA content, and larger, more complex cells with lower Fe^2+^ and higher PHA levels (Figure S3). Our results suggest that cells with a higher intracellular labile Fe^2+^ content typically exhibit a higher magnetosome presence. Moreover, according to FCM population analysis, these high Fe^2+^ cells presented lower PHA levels, supporting the proposed hypothesis of the existence of an energy competition between the magnetosome and PHA production. Nevertheless, further research is required to verify this relationship and to more comprehensively determine the specific conditions that stimulate PHA formation in MTB.

The observed differences in cell size, complexity, intracellular iron concentration, and PHA content among these populations can be attributed to several factors. Variability in the cell cycle and growth phase as well as phenotypic heterogeneity can result in differences in cell morphology and intracellular contents. Environmental microgradients, such as variations in nutrient or oxygen availability, may also create localized differences in bacterial behavior. Furthermore, different stages of magnetosome formation and metabolic imbalances can influence iron and PHA accumulation. Finally, stress responses and genetic diversity within the population can further contribute to the observed heterogeneity.^65^

## 4 Conclusions

The present study explored the effects of iron and oxygen availability on MSR-1 growth, magnetosome biogenesis, and the dynamics of its intracellular iron pool at a multiscale level, to gain further insights into the magnetosome biomineralization process. A correlative microscopy approach was conducted by employing cryoSIM and cryoSXT for the first time in MSR-1 and further complemented with FCM, ICP-OES and TEM. Our findings confirm the critical role of iron for both MSR-1 growth and magnetosome formation. Data obtained from FCM and ICP-OES suggested a potential association in which higher intracellular iron levels facilitate greater magnetosome production. This relationship was demonstrated at the single-cell level by employing correlative microscopy analysis revealing a direct correlation between the labile intracellular Fe^2+^ pool and magnetosome content. This demonstrated that cells exhibiting higher intracellular labile Fe^2+^ concentrations possessed a greater number of magnetosomes in comparison to those with lower iron levels. Additionally, the variation of iron dosages in the media directly impacted both magnetosome chain length and the intracellular iron concentration, with higher extracellular iron concentrations resulting in longer chains and increased iron uptake. We have identified a saturation point at approximately 300 μM iron citrate, beyond which additional iron supplementation did not further increase magnetosome chain length or total intracellular iron concentrations. Moreover, our results also demonstrated that only a fraction of the total intracellular iron content corresponded to magnetite, as the intracellular labile Fe^2+^ pool persisted without depletion throughout the biomineralization process. These findings align with proposed MTB iron uptake models suggesting that iron is first accumulated in the cytoplasm before being transported to the magnetosome vesicles. Overall, these findings highlight the value of single-cell analysis and the necessity of adopting a holistic methodology across different scales to enhance our comprehension of magnetosome biomineralization necessary for efficient future biomanufacturing.

## Supporting Information Available

Iron tolerance test intracellular iron content; CryoSIM images of PG-SK stained MSR-1 cells under various growth conditions; FCM population analysis of PG-SK, Pyr-546, FSC-A, and SSC-A; Correlation between magnetosome content and FCM-PG-SK fluorescence values; Percentage of aerobic and microaerobic cells based on their magnetosome chain length; Time-course evolution of cryoSIM-PG-SK fluorescence; Iron tolerance test Pyr-546 fluorescence values; Supporting movies’ descriptions (PDF)
Movie S1, tomogram of a representative MSR-1 cell containing a long magnetosome chain (AVI)
Movie S2, 3D correlation of CryoSIM and cryoSXT data including 3D segmentation (AVI)
Movie S3, tomogram of representative MSR-1 cells containing short magnetosome chains at the “water window” (AVI)
Movie S4, tomogram of representative MSR-1 cells containing short magnetosome chains at the Fe L_3_-edge (AVI)

## Supplementary Material

am4c15975_si_001.pdf

am4c15975_si_002.avi

am4c15975_si_003.avi

am4c15975_si_004.avi

am4c15975_si_005.avi
