# Supplementary material for: An Integrated Approach to Elucidate the Interplay between Iron Uptake Dynamics and Magnetosome Formation at the Single-Cell Level in Magnetospirillum gryphiswaldense
Source: ACS Appl Mater Interfaces. 2024 Oct 31;16(45):62557–70. doi: 10.1021/acsami.4c15975 (PMC11565563; doi:10.1021/acsami.4c15975)
Supplement: Supplementary file 1 — am4c15975_si_001.pdf [file am4c15975_si_001.pdf]

Supporting information

An integrated approach to elucidate the interplay between iron uptake dynamics and magnetosome formation at the single-cell level in *Magnetospirillum gryphiswaldense*

Marta Masó-Martínez[1,2], Josh Bond[1,2], Chidinma A Okolo[3], Archana C Jadhav[3], Maria Harkiolaki[3,4], Paul D Topham[2], Alfred Fernández-Castané[1,2]*

[1] Energy and Bioproducts Research Institute, Aston University, Birmingham, UK

[2] Aston Institute for Membrane Excellence, Aston University, Birmingham, UK

[3] Beamline B24, Diamond Light Source, Harwell Science and Innovation Campus, Didcot, Oxfordshire, UK

[4] Chemistry department, University of Warwick, Coventry, UK

***Corresponding author email:** a.fernandez-castane1@aston.ac.uk

Supporting Figures

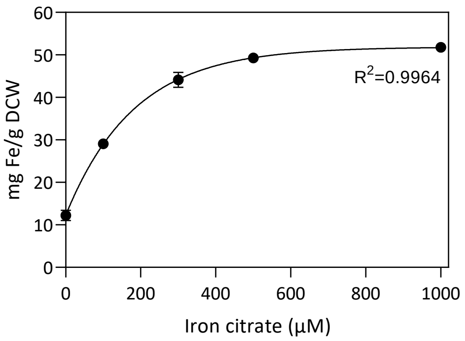

Figure S1. Total intracellular iron concentrations results of MSR-1 iron tolerance test. MSR-1 cells were grown under microaerobic conditions and different iron dosages (0-100-300-500-1000 µM iron citrate). Error bars are standard deviation (n=3).

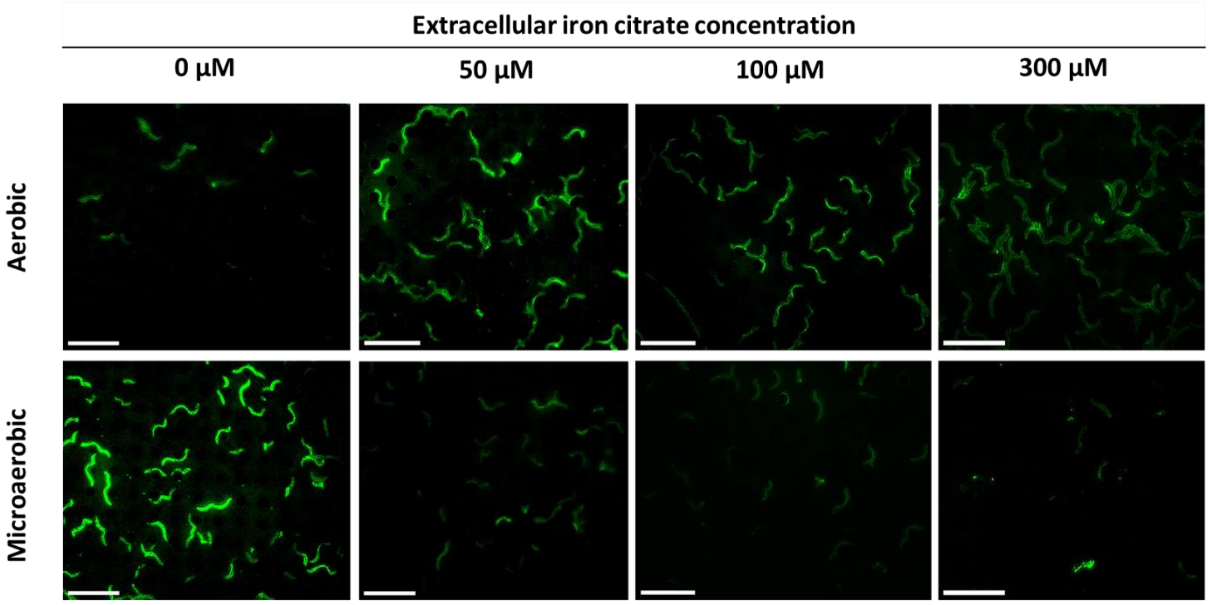

Figure S2. CryoSIM images of *Magnetospirillum gryphiswaldense* MSR-1 cells stained with PG-SK grown under different iron (0 – 50 – 100 – 300 µM iron citrate) and oxygen (aerobic or microaerobic conditions) concentrations. Scale bar = 10 µm.

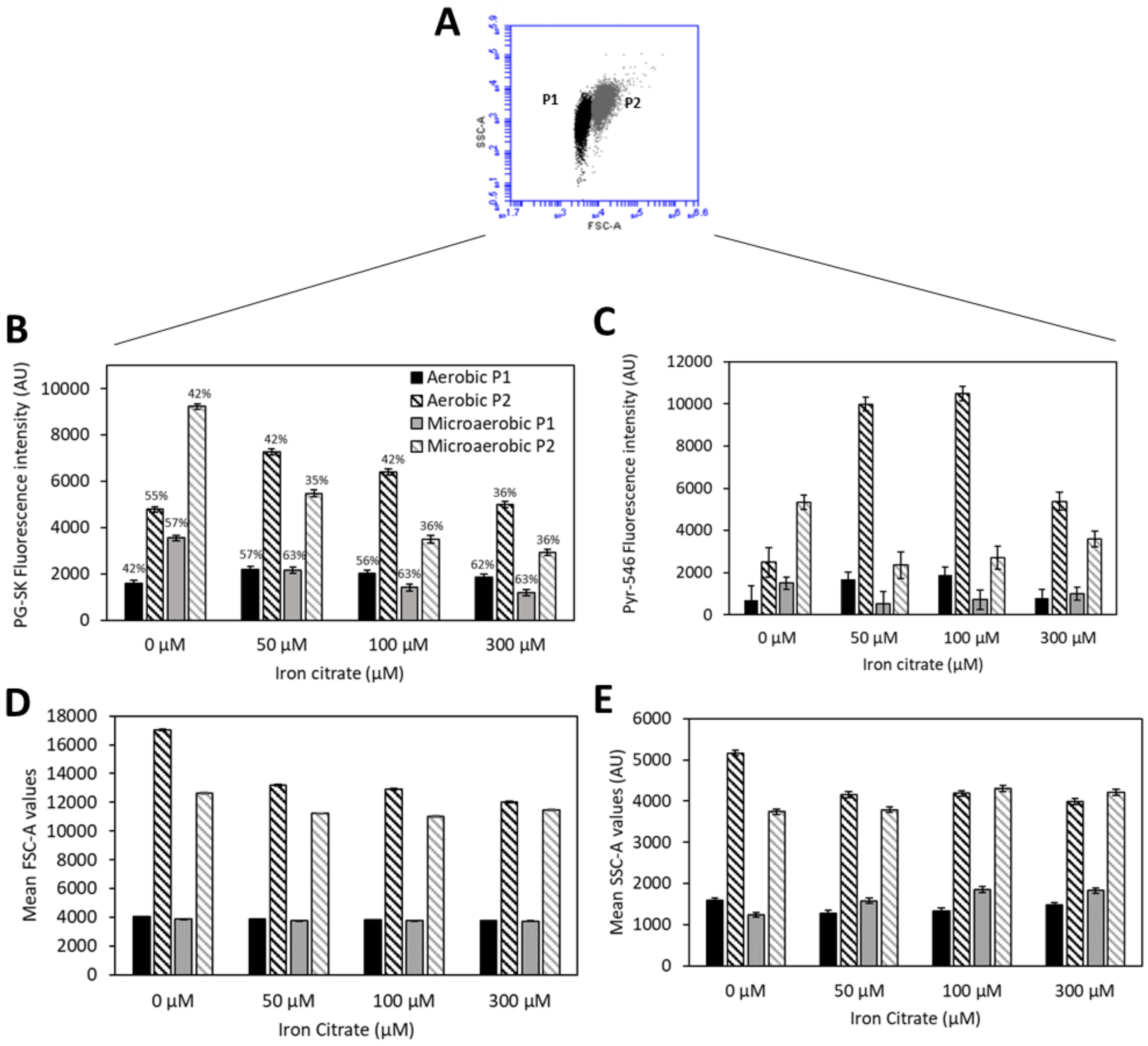

Figure S3. FCM population analysis of MSR-1 cells grown under different iron concentrations (0-50-100-300 µM iron citrate) at microaerobic or aerobic conditions. (A) Representative forward scatter (FSC-A) vs side scatter (SSC-A) dot plot in which two distinct populations (P1 and P2) can be distinguished. For all conditions these two populations could be observed. (B) PG-SK mean fluorescence intensity values. Percentage values represent the size of each population. (C) Pyr-546 mean fluorescence intensity values. (D) Mean FSC-A values. (E) Mean SSC-A values. Error bars are covariance. 25 000 events were analysed per sample by FCM. AU= arbitrary units; P1 = Population 1; P2 =Population P2.

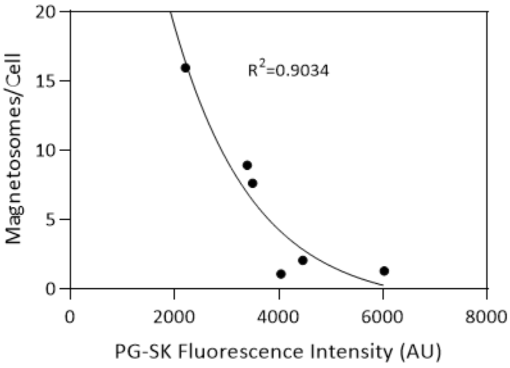

Figure S4. Correlation between mean PG-SK fluorescence values obtained by FCM and the mean number of magnetosome crystals per cell of microaerobic and aerobic MSR-1 cells grown under different iron concentrations (0-50-100-300 µM iron citrate). AU= arbitrary units.

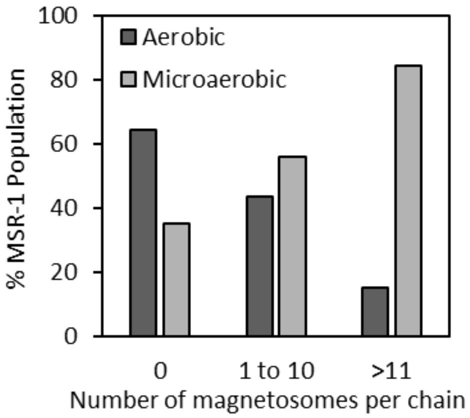

Figure S5. Classification of aerobic and microaerobic MSR-1 cells containing different magnetosome content (long, short or no chain) from Figure 3C (n=104).

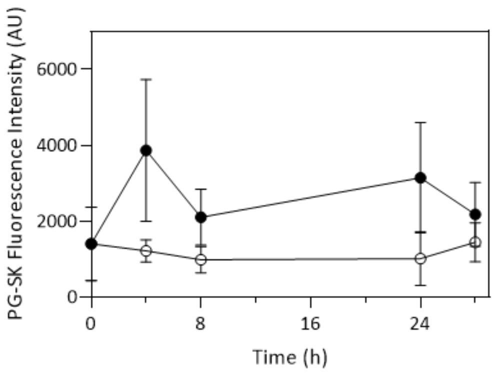

Figure S6. PG-SK mean fluorescence intensity values obtained by measuring fluorescence intensity from cryoSIM data of MSR-1 cells grown under 100 µM iron citrate under microaerobic (white) or aerobic (black) conditions over a period of 28h. AU= arbitrary units.

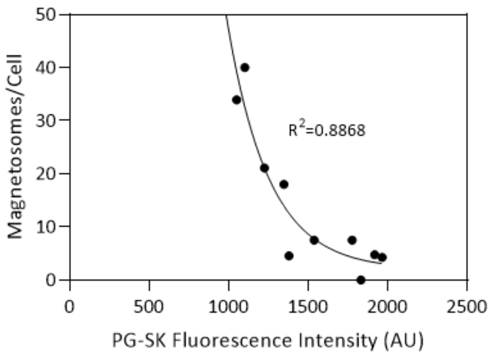

Figure S7. Correlation between mean PG-SK fluorescence values obtained by FCM and the mean number of magnetosome crystals per cell of time-course experiment in which MSR-1 cells were grown under microaerobic or aerobic conditions at a 100 µM iron citrate concentration. AU= arbitrary units.

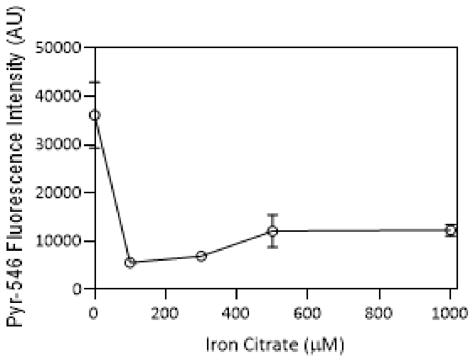

Figure S8. MSR-1 iron tolerance test analysis of PHA content. MSR-1 cells were grown under microaerobic conditions and different iron dosages (0-100-300-500-1000 µM iron citrate) and stained with Pyr-546 to be analyzed by FCM. Error bars are standard deviation (n=3). AU= arbitrary units.

Supporting Movies' Descriptions

Movie S1. Example of a cryoSXT tomogram of a MSR-1 cell containing a long magnetosome chain acquired at 500 eV. Scale bar 2 µm.

Movie S2. CryoSXT tomogram (500 eV) correlated with 3D cryoSIM data of a MSR-1 magnetosome-producing cell. PG-SK was the iron fluorescent probe used. A 3D volumetric representation of this cell is included in which magnetosomes are coloured in pink, PHA granules in blue and the cell membrane in purple.

Movie S3. Example of a cryoSXT tomogram of MSR-1 cells containing a short magnetosome chains acquired at 500 eV. Scale bar 2 µm.

Movie S4. Example of a cryoSXT tomogram of MSR-1 cells containing a short magnetosome chains acquired at 710 eV. Scale bar 2 µm.
